# Bio-Electroanalysis Performance of Heme Redox-Center for *π*-*π* Interaction Bonding of a Methylene Blue-Graphene Modified Electrode

**DOI:** 10.3390/nano13040745

**Published:** 2023-02-16

**Authors:** Porntip Khownarumit, Kanmanee Choosang, Rungtiva P. Poo-arporn, Yingyot Poo-arporn, Narong Chanlek, Werasak Surareungchai

**Affiliations:** 1 Sensor Technology Laboratory, Pilot Plant Development and Training Institute, King Mongkut’s University of Technology Thonburi, Bangkok 10150, Thailand; 2 Biological Engineering Program, Faculty of Engineering, King Mongkut’s University of Technology Thonburi, Bangkok 10140, Thailand; 3 Synchrotron Research and Applications Division, Synchrotron Light Research Institute, 111 University Avenue, Nakhon Ratchasima 30000, Thailand; 4 Faculty of Science and Nanoscience & Nanotechnology Graduate Program, King Mongkut’s University of Technology Thonburi, Bangkok 10140, Thailand; 5 Bangkhuntein Campus, School of Bioresources and Technology, King Mongkut’s University of Technology Thonburi, Bangkok 10150, Thailand; 6 Analytical Sciences and National Doping Test Institute, Mahidol University, Bangkok 10400, Thailand

**Keywords:** heme, electrochemical sensor, methylene blue, graphene, modified electrode

## Abstract

Hemeprotein detection has motivated extensive research on the direct reaction of a heme molecule and a redox dye. The present study used methylene blue as both donor and acceptor for a redox reaction. First, the solid phases of methylene blue (MB) and graphene (GP) formed a π-π interaction bond at the aromatic rings. The conductivity of GP was better than that of carbon in a carbon electrode (CE). Then, the working CE was modified using strong adsorption of MB/GP on the electrode surface. The surface of the electrode was investigated using a modified and an unmodified electrode. The electrode’s properties were studied using voltammograms of redox couple K_3_[Fe(CN)_6_]^3−/4−^. Its reaction was used to find the active area of the modified electrode, which was 1.76 times bigger than that of the unmodified electrode. The surface coverage values of the modified and unmodified electrodes were 8.17 × 10^−6^ and 1.53 × 10^−5^ mol/cm^2^, respectively. This research also studied the application of hemeprotein detection. Hemoglobin (Hb), myoglobin (Mb), and cytochrome c (Cyt. C) were studied by the reaction of Fe (III/II) at the heme-redox center. The electrocatalytic reaction between MB/GP and hemeproteins produced an anodic peak at 0.35 V for Hb, Mb, and Cyt. C. This nanohybrid film enhanced electron transfer between protein molecules and the modified carbon electrode. The amperometric measurements show that the limit of detection was 0.2 µM, 0.3 µM, and 0.1 µM for Hb, Mb, and Cyt. C, respectively. The measurement spanned a linear range of 0.2 µM to 5 µM, 0.3 µM to 5 µM, and 0.1 µM to 0.7 µM for Hb, Mb, and Cyt. C, respectively. Hb showed the lowest sensitivity compared with Mb and Cyt. C due to the role of steric hindrance in the hemeprotein specificity structure. This study offers a simple and efficient fabrication platform for electrochemical sensors for hemeproteins. When compared to other complex immobilization processes, the fabrication method for this sensor has many benefits, including no need for special chemicals and easy preparation and electrode modification—both of which are crucial for the development of electrochemical sensing devices.

## 1. Introduction

**Figure 1:**
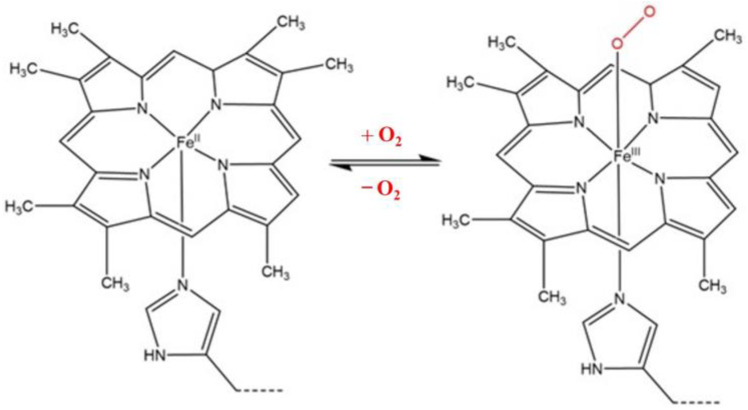
A normal heme complex (Fe protoporphyrin IX) showing the attachment and release of an oxygen molecule.

Heme is a prosthetic group precursor to hemoglobin and is necessary for overseeing the transport of O_2_ and for transferring electrons. The general structure of heme is shown in Figure 1.

Hemeproteins consist of a metalloprotein and an organic compound. The major chemical structure of metalloprotein is an iron–porphyrin group (heteroaromatic ring hosting an iron molecule attached to the nitrogen atom of the porphyrin) [1,2,3], which can transfer electrons in the metabolic pathway of a biological system. The organic compound can consist of amino acid groups linked into chains by peptide bonds [4].

There are several biologically important kinds of heme; however, three of them have been commonly studied: hemoglobin (Hb), myoglobin (Mb), and cytochrome c (Cyt.C). These hemeproteins can be found in different parts of the body, for example, in the blood (Hb), in muscle cells (Mb), and in mitochondria (Cyt. C), among others. They have different structures and therefore perform different functions.

Hb is bound with 4-heme (4-Fe (III)) of hetero-tetrameric molecules and 4 polypeptide chains and has a molecular weight of 64.5 kDa. Thus, the chemical and physical behaviors of Hb are affected by iron bearing in each chain-molecule heme. The allosteric behaviors are related to the heme redox-center transfer [5,6,7].

Mb is bound with 1-heme (1-Fe (III)) of a cytoplasmic molecule that is a hemeprotein in cardiac myocytes tissue and oxidative skeletal muscle fibers. Mb has a molecular weight of 17.05 kDa. Mb is related to the storage of oxygen in muscles and the removal of nitric oxide to protect myocyte respiration. It is especially abundant in the heart and skeletal muscles of diving mammals such as whales and seals, which allows them to hold their breath for long periods of time [8,9].

Cytochromes are the smallest redox-active molecules containing a heme with an Fe atom at their center as a cofactor. The International Union of Biochemistry and Molecular Biology (IUBMB) referred to four cytochrome types composed of different biding modes in hemes. These are cytochrome a, cytochrome b, cytochrome c, and cytochrome d [10]. Other types can be found in the biochemical literature such as cytochrome o and cytochrome P450. Cyt. C was used in this research because it consists of a small chain of 1-polypeptide and one atom of Fe in the heme, which produces reduction potentials.

In medicine, Hb screening tests obtained from urine or blood samples are used to diagnose various diseases associated with hemoglobin disorders such as anemia [11], hematemesis [12], hematuria [13], hemoglobinuria [14], and diabetes [15]. In Thailand, three protocols for the detection of Hb are currently used in hospitals, MCV (mean corpuscular volume) [16,17]; KKU-OF (Khon Kaen University (KKU), a modified one-tube osmotic fragility test); and KKU-DPIP (Khon Kaen University), a modified dichlorophenolindophenol (DCIP) precipitation test [18,19,20]. If a test produces abnormal results, PCR analysis followed by other techniques must be performed, which include absorption peaks [21], fluorimetry [22], colorimetry [23], HPLC [24], chemiluminescence [25], and the spectrophotometric method [26], the latter of which can be used to detect Hb and Mb. Although these techniques provide good qualitative and quantitative results, they require trained operators and complicated measurements.

The detection of Hb can be also used to diagnose hematopoietic organs, hemolysis, and anemia. This can be done using colorimetric detection kits with blood samples. Normal levels are 13.8 g/dL to 18 g/L (8.56 mmol/L to 11.17 mmol/L) in men, 12.1 g/dL to 15.1 g/dL (7.51 mmol/L) in women, 11 g/dL to 16 g/dL (6.83 mmol/L to 9.93 mmol/L) in children, and 11 g/dL to 14 g/dL (6.83 mmol/L to 8.69 mmol/L) in pregnant women [27,28]. The detection of Mb can be used to evaluate muscle damage in patients with Rhabdomyolysis. Mb sampling is received from urine. Mb is measured by applying the ELISA method to a urine sample [29]. Cyt. C has been related to apoptosis [30] and mitochondrial abnormalities producing muscle weakness [31]. Cyt. C is normally detected using the ELISA technique with serum samples [32].

However, the above method is not convenient for screen testing because toxic and carcinogenic substances are used to prepare a measurement solution along with an extraction solvent, and a highly purified reagent is required. Therefore, a well-trained user is necessary to manage the instrument. In addition, it is time-consuming due to sample pretreatment and the long reaction time.

An alternative analysis is possible using a modified electrode. The analysis of Hb is compared with Mb and Cyt. C. Analysis and an early monitoring report should be used to diagnose abnormal hemeproteins, similar to a screening test before a definitive diagnosis is conducted at the hospital.

Electrochemical sensors provide a simpler technique. One group of researchers has studied the use of hemeprotein films immobilized on an electrode surface to fabricate biosensors, biomedical devices, and enzymatic bioreactors [33] using the hemeprotein films immobilized on an electrode surface. Other film options include hydrogel polymers [34], polyelectrolytes [35], and clay composite films [36]. These researchers were interested in a model of electron transfer between enzymes in real biological systems, for example, Cyt. C reductase immobilized on carbon paste electrode [37] and hemeproteins immobilized on gold nanoparticles [38]. Both studies investigated the electrocatalytic effect of Cyt. C, Mb, and Hb. However, the electro-reaction process of direct electron transfer does not occur easily at the surface of a conventional electrode because the behavior of the three-dimension structures of hemeproteins hinders interaction and produces denaturation adsorption or passivation on the electrode surface [39].

This research focuses on the electrocatalytic reaction of hemeprotein. Nanohybrids were used to modify the surface of screen-printed electrodes. Earlier works have used MB/CNTs to modify glassy electrodes [40]. To our knowledge, while there are numerous studies on employing graphene oxide, there are relatively few papers on the use of methylene blue/graphene nanohybrids for electrochemical sensing. Li, Y. et al. (2011) [41] utilized anti-CEA/AuNPs/MB/Gp-Nf-modified GCE as an immunosensor to detect carcinoembryonic antigen, and Qiao, L. et al. (2014) [42] used BSA/anti-chlorpyrifos/GS-MB/GNPs-modified GCE to detect chlorpyrifos. The use of a composite of graphene and methylene blue with three different particle sizes of bulk graphene flakes (BGF), graphene flakes (GF), and graphene quantum dots (GQD) as a humidity sensor array was another application [43]. Since GO lacks an aromatic structure that aids in electron delocalization through their structure and is less effective than Gr in the event of generating potent π–π interactions with MB. Additionally, we selected GP because the nanohybrid MB/GP has the advantage of being richer in π-π bonding than CNTs [44]. The development of a disposable electrochemical sensor for the direct detection of Hb, Mb, and Cyt. C with an easy and low-cost preparation method is described in this study for the first time.

This research aims to compare the redox catalysis of heme that can occur due to the electrocatalytic reaction on the MB/GP-modified electrode. In the experiments, three hemeproteins (Hb, Mb, and Cyt. C) were measured by a screen-printed electrode modified with nanohybrids of MB/GP. This study concerns the steric affected molecule that responds to the electrochemical measurement (EC). In this investigation, a disposable electrode was designed that allows low-cost and rapid testing. No polishing is required to clean the electrode surface, which also reduces the preparation time compared with earlier works. The results obtained in this study are useful to develop a protocol for Linear sweep voltammetry (LSV) in portable equipment. Moreover, this work can be beneficial for pre-diagnostic or screening before using a more sophisticated and expensive instrument.

This study provides not only the crucial details about the redox catalysis of three different hemeproteins on the MB/GP-modified electrode, but it also offers a useful electrochemical sensor for the quick and painless detection of hemeproteins. In comparison to conventional approaches, our sensor is substantially easier to use, less expensive, and capable of being widely used for early detection of abnormal hemeprotein levels. This is because of their ease of fabrication, minimal solvent and reagent usage, and a high rate of production. The benefits provided by this potential sensor technology for monitoring the concentration of hemeproteins in medical analyses may be employed for various sorts of other significant biomarkers for numerous diseases.

## 2. Materials and Methods

### 2.1. Reagents

Human hemoglobin (MW 64,500 Da) and horse heart myoglobin (MW 17,600 Da), cytochrome c (MW 13,000 Da) were purchased from Sigma-Aldrich Pte. Ltd. (Merck). (Shanghai, China). Methylene blue was obtained from Merck (Shanghai, China). Graphene (size 20 nm–100 nm) was obtained from Cheap Tubes, (Cambridgeport, VT, USA). The remaining chemicals were of analytical grade. All reagents were used without further purification. Phosphate buffer solutions (0.1 M PB pH 7) were prepared by mixing a stock of standard solution 0.1 M Na_2_HPO_4_ and 0.1 M NaH_2_PO_4_, and later by adding 0.1 M KCl (from Sigma-Aldrich Pte. Ltd. (Merck), (Shanghai, China). All solutions were deoxygenated by bubbling high-purity nitrogen for at least 10 min.

### 2.2. Preparation of Nanohybrid Materials

Ten milligrams of MB and two milligrams of GP were mixed in 10 mL distilled water and then the mixtures were sonicated for 4 h at room temperature. The resulting suspension was filtered with a porous filter (0.45 µm, Millipore, Burlington, MA, USA). The final pellets of MB/GP were first thoroughly rinsed with distilled water to remove non-absorbed MB. The total weight of MB/GP, 2 mg, was dispersed in 250 mL distilled water.

### 2.3. Fabrication of Electrode

Each screen-printed electrode was cleaned with distilled water and dried with high-purity nitrogen. Meanwhile, the stock of 2 mg MB/GP nanohybrids was dispersed in 250 mL of distilled water. Finally, 4 µL of a 6.25% solution containing a volume fraction of nanohybrids was deposited onto the working electrode surface (area of 2 × 3 mm^2^), and then it was dried at 80 °C for 30 min.

### 2.4. Instrumentation and Procedure

Cyclic voltammetry and linear sweep voltammetry were performed using a potentiostat (Autolab PGSTAT128N, Eco Chemie, The Netherlands). With its control software (NOVA Eco Chemie), a three-electrode cell was used, which consisted of a carbon screen-printed electrode (area of 2 × 3 mm^2^, Quasense. Co., Ltd., Bangkok, Thailand) performing as a working electrode, a platinum wire used as a counter electrode (length of 5.7 cm), and an Ag/AgCl electrode in saturated KCl (NF343 RF-1B, ALS Co., Ltd., Tokyo, Japan), used as a reference electrode. Cyclic voltammetry of MB/GP film was carried out using a phosphate-buffered solution containing no hemeproteins. Each of the electrodes was kept in the desiccator until being used for evaluation in the next experiment. The buffer solution was purged with high-purity nitrogen for 30 min before each experiment. X-ray photoelectron spectroscopy (XPS, PHI VersaProbe II) was carried out at the SUT-NANOTEC-SLRI Joint Facility, BL5.2: SUT-NANOTEC-SLRI, Synchrotron Light Research Institute. The excitation energy was 1486 eV using an Al Kα source. The morphology of MB/GP nanohybrids was characterized by using a scanning electron microscope (FE-SEM) (NovaTM NanoSEM 450), (JEM-1011, JEOL, Tokyo, Japan) operated at 10.0 kV, with a magnification of 2500× and 10,000×. Ultraviolet–visible (UV-Vis) absorption spectra of MB in aqueous solutions were detected with a UV-Vis spectrophotometer (CYT5FV, Agilent BioTek, San Diego, CA, USA). The characteristic functional groups of MB/GP nanohybrids were examined using Fourier transform infrared spectroscopy (INVENIO, Bruker, Germany). The zeta potentials of MB/GP nanohybrids were measured using electrophoretic light scattering (ELS) spectrophotometer (Dynamic Light scattering model Zetasizer Nano ZS, Malvern, England).

*Ee* = *E°’* + (*RT*/*nF*) *ln* [(*Fe*(*CN*)_6_)^4−^]/[(*Fe*(*CN*)_6_)^3−^] (1)

*E* = *E°’* – (0.05916/*n*) *log* [(*Fe*(*CN*)_6_)^4^*^−^*]/[(*Fe*(*CN*)_6_)^3^*^−^*] (2)

Δ*Ep* = *|Ep^ox^* − *Ep^red^|* = 0.059 *V*/*n* (3)

*i* = *AFJ* (4)

*I_p_* = 0.4463 *×* (*F*^3^/*RT*)^1/2^
*× n*^3/2^
*× A*_0_ × *D*_0_^1/2^
*× C × ν*^1/2^ (5)

*I_p_* = (–3.26 × 10^–5^ (*A*s^1/2^/*V*^1/2^)) × (*ν*^1/2^(*V*/*s*)^1/2^) – (4.827 × 10^–5^ (*A*)), *R*^2^ = 0.97 (6)

*I_p_* (*Amp*) = (−5.73 × 10^–5^ (*As*^1/2^/*V*^1/2^)) × (*ν*^1/2^(*V*/*s*)^1/2^) − (7.164 × 10^–5^ (*A*)), *R*^2^ = 0.92 (7)

*I_p_* = (*n*^2^*F*^2^*ΓA*_0_*ν*)/(4*RT*) (8)

*I_p_* (*Amp*) = (−6.638 × 10^−5^ (*As*/*V*)) × *ν* (*V*/*s*) − 1.450 × 10^–5^ (*A*), *R*^2^ = 0.96 (9)

*I_p_* (*Amp*) = (−3.762 × 10^−5^ (*As*/*V*)) × *ν* (*V*/*s*) − 1.270 × 10^–5^ (*A*), *R*^2^ = 0.93 (10)

## 3. Results

### 3.1. Characterization of MB/GP on Modified Electrode

**Figure 2:**
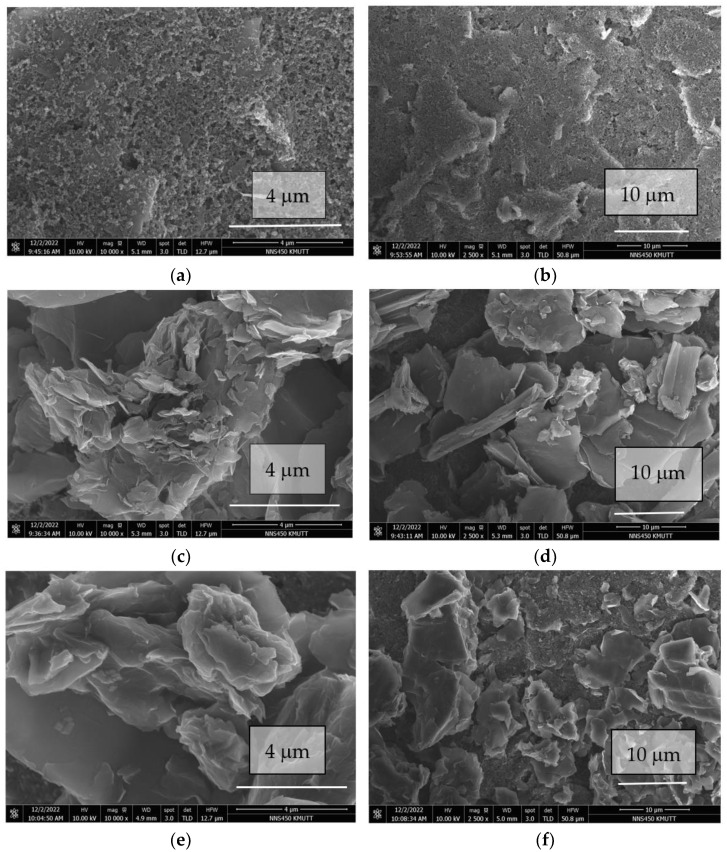
SEM images of unmodified SPCE (**a**,**b**), modified MP/GP nanohybrids on SPCE (**c**,**d**), and modified MP/GP nanohybrids on SPCE that was scanned by CV for 100 cycles (**e**,**f**).

The morphologies of the prepared MB/GP nanohybrids were characterized by a scanning electron microscope (SEM). The modified electrode SEM image, as seen in Figure 2c–f, exhibits a characteristic layered sheets structure with the morphology of flake-like forms randomly packed in stacked structures, indicating that the MB/GP nanohybrids successfully attached or doped on SPCE tightly. An SEM image of an electrode with an unmodified surface and no GP flake-like structures is shown in Figure 2a,b. The MB/GP nanohybrids were still embedded onto the SPCE surface as shown in SEM images before CV was fun, showing that the nanohybrids were still intact after the modified electrode underwent 100 cycles of CV (Figure 2e–f).

**Figure 3:**
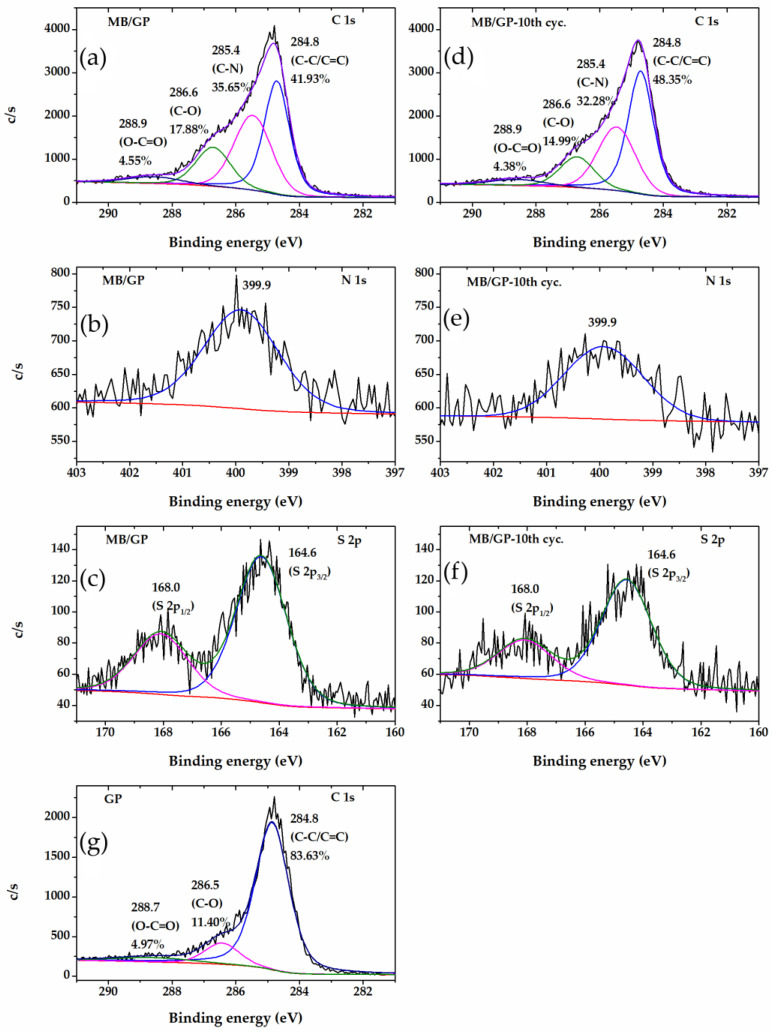
The XPS spectra of MB/GP (**a**) C 1s, (**b**) N 1s, (**c**) S 2p, MB/GP at 10th cycle of CV scan (**d**) C 1s, (**e**) N 1s, (**f**) S 2p, and GP (**g**) C 1s.

X-ray photoelectron spectroscopy of MB/GP before and after the 10 cycles of CV scanning was measured to achieve insight into the adsorption capacity of MB on the produced electrode. Three peaks that matched the C-C (284.8 eV), C-N (285.4 eV), C-O (286.6 eV), and O-C=O (288.9 eV) peaks were fitted into the C 1s XPS spectra of MB/GP (Figure 3a) [45,46]. Due to contributions from nitrogen species, a fine scan for N 1s revealed a broad and symmetric signal (Figure 3b). Peak fitting analysis revealed binding energies of 399.9 eV, which were attributed to the C-N groups [47]. In the S 2p spectra shown in Figure 3c, two sulfur-related peaks were found at 164.6 eV (S 2p_3/2_) and 168.0 eV (S 2p_1/2_). These two peaks should be assigned the sulfur of the phenothiazine structure in MB [48]. As a result, the XPS data suggested that MB was successfully adsorbed onto the electrode. After the 10th cycle of CV scanning, the C-1s, N-1s, and S-2p XPS spectra of the MB/GP-10th cycle were well matched to that of the as-prepared electrode, suggesting that the MB is stable under CV measurement (Figure 3d–f).

Graphene is a honeycomb-shaped single atomic layer of SP_2_-hybridized carbon atoms. By π–π stacking interaction, the aromatic molecules may be adsorbed on its hexagonal nanostructure, forming a stable hybrid structure and improving their electrical characteristics [49]. As seen in Figure 3g, the primary component of C-C/C=C and two minor C-O and O-C=O species were both visible in the GP C 1s XPS spectrum. As a result, GP has a large number of π electrons. These findings imply that π-π interactions comprise the majority of the interactions between GP and MB. However, a tiny number of carboxyl groups at the edges of the carbon sheet produce a negatively charged surface that also facilitates the electrostatic adsorption of positively charged MB molecules.

**Figure 4:**
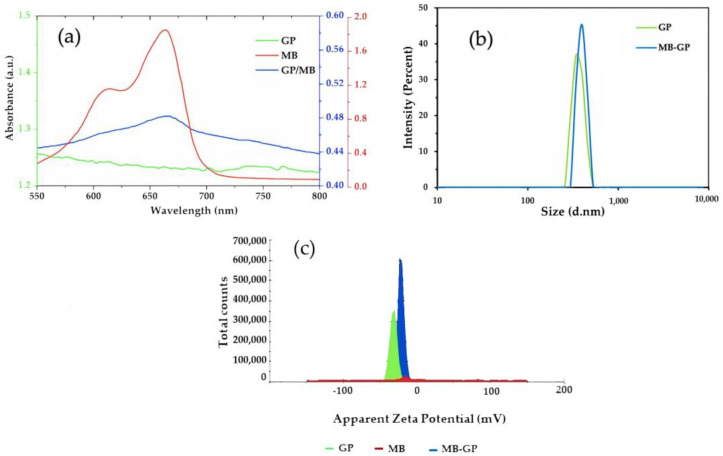
(**a**) UV spectra of MB (red), GP (green), and MB/GP nanohybrids (blue) in DI water. (**b**) Particle sizes of GP (green) and MB/GP nanohybrids (blue) measured by DLS. (**c**) Zeta potential of MB (red), GP (green), and MB/GP (blue) nanohybrids measured by ELS.

UV-vis spectroscopy was used to examine the formation of the MB/GP nanohybrids. The UV-vis spectra of MB, GP, and MB/GP in the aqueous solution are shown in Figure 4a. The strong adsorption of MB onto the GPs was observed through the large decrease in the UV-vis absorbance of the MB solution upon the dispersion of the GPs into the solution [49]. Free MB in the aqueous solution has a significant absorbance at 664 nm (green), which is typical of the MB monomer in the solution. Additionally, a shoulder peak at 610 nm was detected and assigned to the MB dimer in an aqueous solution [50]. The spectra of the MB/GP nanohybrids (purple), which resemble that of free MB, demonstrated the chemisorption of MB onto the GP. Extensive analysis of the spectra of free MB and MB/GP nanohybrids indicates that the absorption peak of the latter was shifted to 662 nm after the synthesis of MB/GP. The absorption bands of the GO-MB and CNTs-MB in water were previously known to be around 663 and 646 nm. The shift was attributed to GO-MB and CNTs-MB interactions, implying that the electronic conjugation structure inside the sheets was extended during the alteration process [50,51,52].

DLS analysis was performed to estimate the size and surface charge of the MB, GP, and MB/GP nanohybrids, as shown in Figure 4b,c. The particle sizes of GP and MB/GP nanohybrids measured by results of DLS were 363.7 and 396.4 nm. Additionally, zeta potential analysis revealed that the MB, GP, and MB/GP nanohybrids possessed negative charges of −28.9, −31.8, and −21.5 mV, respectively. This high negative value of zeta potential can be attributed to binding force among the agglomerated nanoparticles [53], suggesting that small particles in suspension resist aggregation and tend to disperse homogeneously in the solution. These findings can attest to MB’s binding to GP.

**Figure 5:**
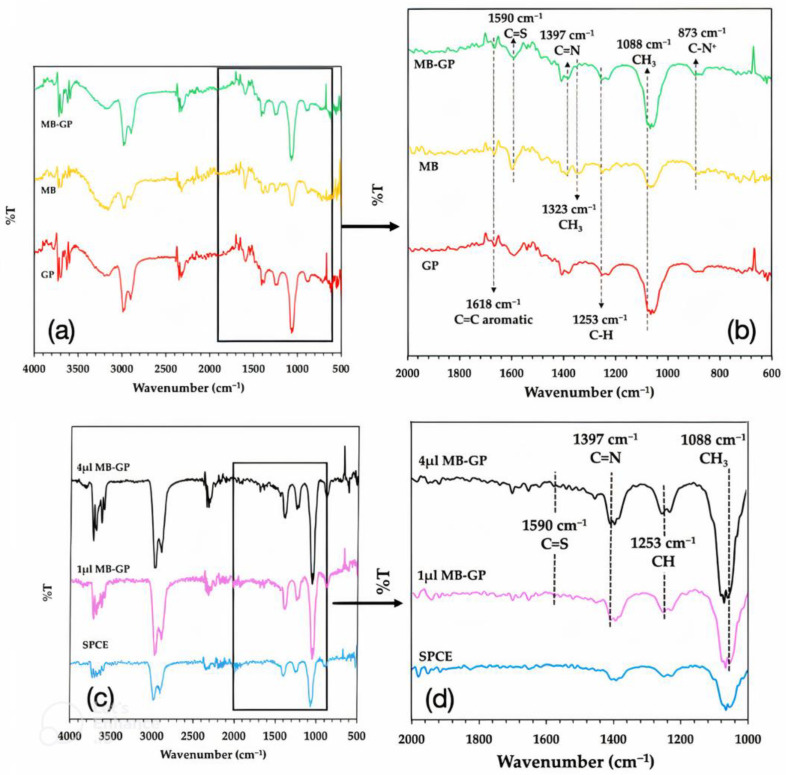
FT-IR spectra of GP (bottom), MB (middle), and MB/GP (top) in DI water (**a**) at 500–5000 cm^−1^ and (**b**) at 600–2000 cm^−1^. FT-IR spectra of SPCE (bottom), 1 μL of Mb/GP (middle), and 4 μL of Mb/GP (top) modified on SPCE (**c**) at 500–4000 cm^−1^ and (**d**) at 1000–2000 cm^−1^.

The FTIR spectroscopy was used to characterize the GP, MB, and MB/GP nanohybrids at 400–4000 cm^−1^ to obtain their spectra. Figure 5a,b show the spectra of GP, MB and MB/GP in DI water. The spectra of GP, MB, and MB/GP that were modified on SPCE are displayed in Figure 5c,d. The apparent peaks of 1618 cm^−1^ (C=C aromatic ring), 1590 cm^−1^ (C=S), 1397 cm^−1^ (C=N), 1253 cm^−1^ (CH in plane), and 1088 cm^−1^ (CH_3_) [54] can be seen in the FT-IR spectrum of MB in DI water (Figure 5b, middle). The spectrum of GP in DI water (Figure 5b, bottom) exhibits identical peak positions for the C=C aromatic ring, CH in plane, and CH_3_ at 1618 cm^−1^, 1253 cm^−1^, and 1088 cm^−1^, respectively [55]. The FT-IR spectrum of MB/GP nanohybrids in water (Figure 5b, top) shows a C=C aromatic ring from unoxidized sp^2^ at 1618 cm^−1^, C=S from MB at 1590 cm^−1^, CH_3_ at 1323 cm^−1^, CH in plane at 1253 cm^−1^, CH_3_ at 1088 cm^−1^, C=N at 1397 cm^−1^, and C-N^+^ (symmetric stretching) at 873 cm^−1^.

Compared to the pure GP, it was observed that many new peaks are imported into the FTIR spectra of the GP after MB adsorption. The new peaks of C=S at 1590 cm^−1^ CH_3_ at 1323 cm^−1^ and C=N at 1397 cm^−1^ are consistent with those identified in the MB spectra, while these peaks cannot be found in the GP. Additionally, after the MB’s adsorption onto the GP, its peaks at 1397 cm^−1^ and 1323 cm^−1^ partially vanished. This finding shows that a certain number of MB molecules were absorbed by the GP.

Then, as shown in Figure 5c,d, FTIR measurements of the GP, MB, and MB/GP nanohybrids modified on SPCE were also performed. When the concentration of MB/GP nanohybrids on the electrode surface is increased, the spectrum of 1 μL and 4 μL of MB/GP nanohybrids modified on SPCE clearly demonstrated an increase in the magnitude of % transmittance. This indicates that the concentration of MB on the electrode surface grew along with the amount of MB/GP.

### 3.2. The Electrochemical Behavior of the Electrode

**Figure 6:**
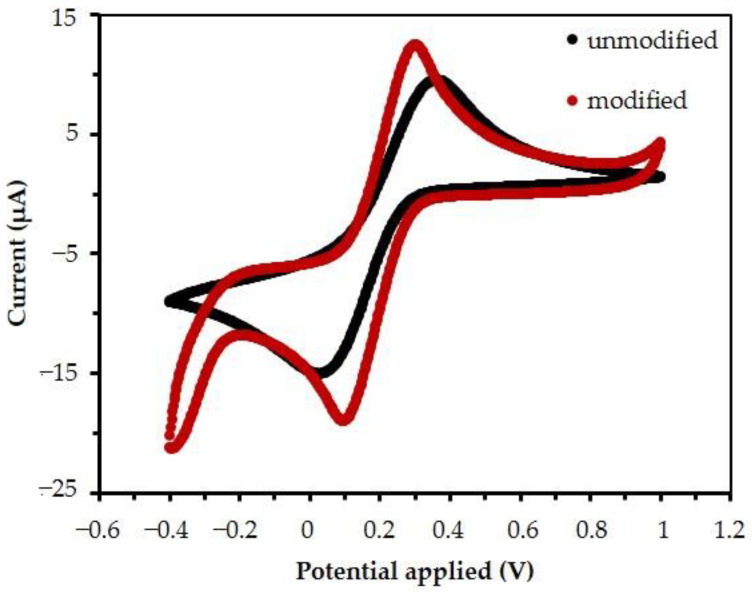
Cyclic voltammogram of the modified and unmodified electrode in 5 mM K_3_Fe(CN)_6_ and adding 0.1 M KCl scanned at 20 mV/s.

The working electrode was tested before and after modification with MB/GP with cyclic voltammetry using a solution of 5 mM of K_3_(Fe(CN)_6_)^3−/4−^ and 0.1 M KCl. In Figure 6, the forward scan of the unmodified electrode (0.4 to 1.2 volts) shows that the oxidation state of (Fe(CN)_6_)^3−^ accepts an electron from the electrode and becomes reduced to (Fe(CN)_6_)^4−^. Then, the (Fe(CN)_6_)^4−^ molecule transfers an electron to the electrode during the reverse scan of an unmodified electrode, oxidizing the molecule at the electrode surface. This electrolysis reaction was related to the Nernst equation, for which the standard potential (*E*°’) was established under the equilibrium potential (*Ee*) of [(Fe(CN)_6_)^4−^]/[(Fe(CN)_6_)^3−^]. This is given by Equation (2). The heights of the forward and reverse current peaks have the same magnitude and peak-to-peak separation, which is independent of scan rate and was about 0.059 V (at 25 °C). This indicates the reversible electrode process and is given by Equations (1)–(3) [56], where *F* is Faraday’s constant (96,480 C/mol), *R* is the gas constant (8.314 J/kmol), *T* is the temperature (298 K), and *n* is the number of exchanged electrons.

At the forward scan, the peak potential (*Ep^ox^*) of the unmodified electrode is +0.32 volts. When the modified electrode MB^2+^ can react with [(Fe(CN)_6_)^3−^], then *Ep^ox^* of the modified electrode shifts to +0.29 volts, and the current response is increased by the flux (*j*) of [(Fe(CN)_6_)^3−^], reaching the electrode surface (moles cm^−2^ s^−1^). This is related to active area (*A* (cm^2^ s^−1^)). This current response (*i*) is given by Equation (4) [56].

As shown in Figure 6, the voltammogram for both electrodes displays the redox species undergoing quasi-reversible electrode reactions. The results show that the delta values between anodic and cathodic peaks were 0.19 V and 0.33 V for the unmodified and modified electrodes when compared with Δ*Ep* of Equation (4). This suggests that the total active area of the modified electrode increases, as will be confirmed later.

### 3.3. Total Active Area

The reaction areas of the unmodified and MB/GP-modified working electrodes were compared. The active area of an electrode and the reactivity of redox species at its surface can both be controlled by adjusting scan rate, according to the Randles–Evk equation [56,57,58,59,60]. This is according to Equation (5), where *I*_p_ corresponds to the peak current (measured in amperes), *F* is Faraday’s constant (96,480 C/mol), *R* is the gas constant (8.314 J/kmol), *T* is the temperature (298 K), *n* is the number of exchanged electrodes, *A*_0_ is the total active area (cm^2^), *D*_0_ is the diffusion coefficient (*D*_0_ = 7.6 × 10^−6^ cm^2^s) [49,50], ν is the scan rate (V/s), and *C* is the concentration (5 × 10^−3^ mol/cm^3^).

**Figure 7:**
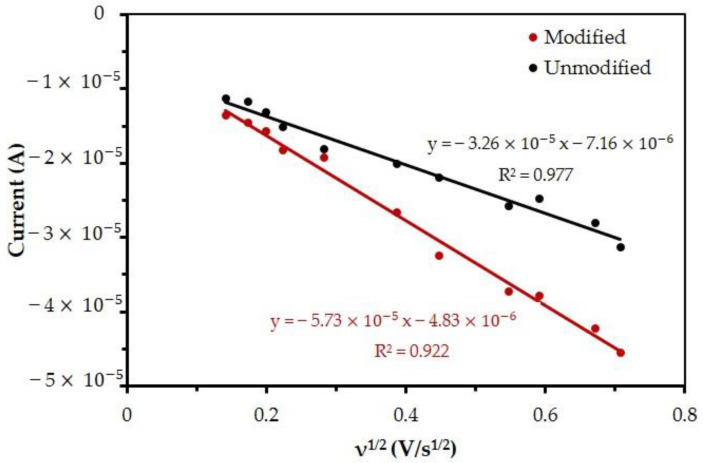
Measurement of oxidation peak current, for unmodified and modified electrodes, as a function of the applied scan rate. The measurements were obtained using a solution of 5 mM K_3_[Fe(CN)_6_] and 0.1 M KCl. This graph was used for the calculation of the total active area.

Figure 7 shows that the cathodic peak currents at both the unmodified and modified electrodes depend on the square root of the scan rate, as is the case in the Randles–Ševčík equation. Linear regressions were also plotted for the data obtained with unmodified and modified electrodes, which correspond to Equations (6) and (7), where R^2^ is the coefficient of determination.

Using Equations (6) and (7) and the known values for the remaining constants, one can calculate the total active areas for the unmodified and modified electrodes which equal 8.71 × 10^−6^ cm^2^ and 1.53 × 10^−5^ cm^2^, respectively. This shows that the active area of the modified electrode is 1.76 times larger than that of the modified electrode.

### 3.4. The Surface Coverage

In a similar fashion, the surface coverage of the electrodes can be obtained from plots of peak current versus square root of the scan rate by using the equation of voltammetry for a Nernstian adsorbate layer (Equation (8)) [59], where *Γ* is the surface coverage (mol/cm^2^) and the total active area was obtained from the previous calculation.

**Figure 8:**
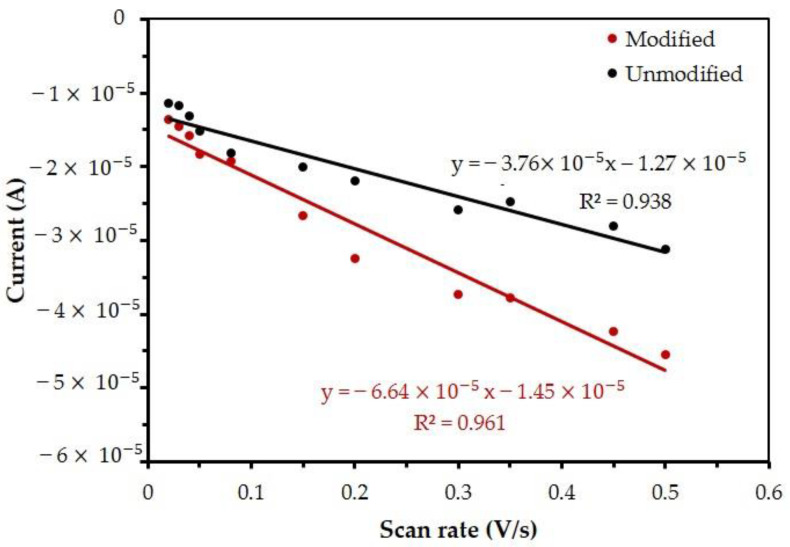
Measurements of cathodic peak current for unmodified and modified electrodes as a function of the applied scan rate. The measurements were obtained using a solution of 5 mM K_3_[Fe(CN)_6_] and 0.1 M KCl. This graph is used for the calculation of surface coverage.

Figure 8 shows the oxidation peak current at both the unmodified and the modified electrodes as a function of the scan rate. As before, linear regression for the cases of unmodified and modified electrodes was obtained, leading, respectively, to Equations (9) and (10).

According to Equations (9) and (10) for the unmodified and modified electrode, surface coverage (*Γ*) is calculated to follow Equation (4) which then obtained *Γ* is 4.5 × 10^−6^ mol/cm^2^ and 7.94 × 10^−6^ mol/cm^2^, respectively. This result suggest that the modified electrode could better catalyze the reaction of Hb, Mb, and Cyt. C, since heme would have more chances of finding Mb/GP per unit area of electrode.

### 3.5. Stability of MB/GP Film on SPCE

**Figure 9:**
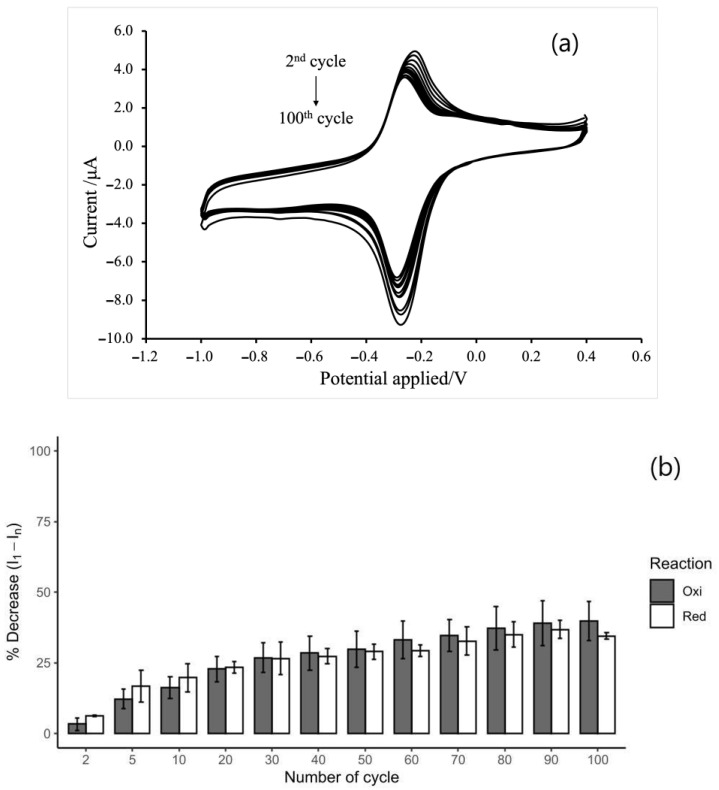
(**a**) Cyclic voltammograms of the MB/GB-modified electrode SPCE on scanning 100 cycles in 0.1 M PBS pH 7.0 at a scan rate of 20 mV/s. (**b**) Plots of the percentage decrease in redox current response from the initial response over n cycles (n = 2, 5, 10, 20, 30, 40, 50, 60, 70, 80, 90, 100).

The stability of the modified electrodes was then examined using cyclic voltammetry, which involved scanning in 0.1 M PB with 0.1 M KCl from the first to the hundredth cycle (*n* = 3) at a scan rate of 20 mV/s. Figure 9a depicts a cyclic voltammogram of an electrode with MB/GP modifications at a scan rate of 20 mV/s, with the electrode being scanned for up to 100 cycles. After performing continuous scanning for 100 cycles, we observed a slight decrease in the redox peak current. As can be seen in Figure 7B, on the first cycle, the percentage decrease in redox peak current of MB/GB-SPCE was accepted as 0% of the reduction peak current for the 20th cycle decreased less than 3% of the response for the 10th cycle, and for each further 10th cycle, the current response also decreased less than 3%. It was considered that the steady background current be shown at the tenth cycles. As a result, CV scans were conducted prior to performing each electrocatalytic reaction in subsequent experiments. After 100 cycles, the reduction and oxidation currents remained at 85% and 76% of the response from the 10th cycles, respectively. This indicates that the electrode containing the MB/GP nanohybrid was strongly attached to the surface of SPCE and that the modified electrode possessed good stability.

### 3.6. Electrocatalytic Reaction of Hemeproteins on MB/GP Modified Electrode

**Figure 10:**
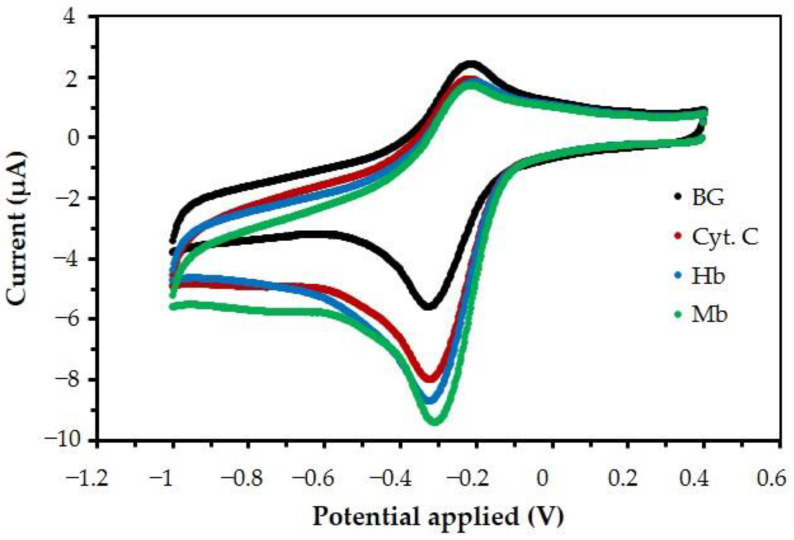
Cyclic voltammograms (10th scan cycles) at 20 mV/s in pH 7 buffers of (black) an MB/GP-modified electrode without hemeproteins and when adding 0.5 μM of (dark green) Hb, (light green) Mb, and (red) Cyt. C.

The solution containing a volume fraction of 6.25% (*v*/*v*) of MB/GP nanohybrids was used to modify the screen-printed electrode. A pretreatment step was applied to the modified electrode to prepare a stable background current response. After that, a hemeprotein sample was added to the buffer solution. Only the 10th scan cycles of the voltammograms were used for the measurements. The voltammograms shown in Figure 10 present an evident catalytic current after hemeprotein samples were added into the buffer solution. The measurements suggest good reversibility of the electrode reactions and no detachment of the MB/GP film from the electrode surface.

After the forward scan of the voltammograms, quasi-reversible electrode reactions occurred. The catalytic potential peaks appeared at −0.35 V versus the Ag/AgCl reference electrode, which was observed for Hb, Mb, and Cyt. C. The scan rate of 20 mV/s shows that *I*_o_ (−2.336 μA) is the reduction current of the MB/GP = modified electrode which was measured (at the 10th scan cycle) in buffer solution and that *I*_n_ is the reduction current of −6.722 μA (Hb), −7.351 μA (Mb), and −5.983 μA (Cyt. C) at the MB/GP-modified electrode. Therefore, the ratios of *I*_n_/*I*_o_ of 0.5 μM of Hb, Mb, and Cyt. C are equal to 2.651, 2.899, and 2.359, respectively. After the potential was applied to the working electrode, oxidized MB was converted to its reduced form [61,62]. Once the hemeproteins diffused to the surface of the electrodes, they reacted by exchanging electrons with the Mb/GP film, converting hemeproteins.

**Figure 11:**
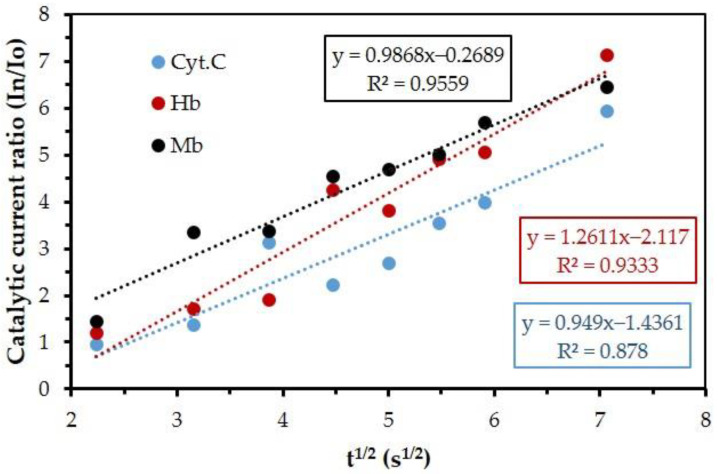
Catalytic current ratio (I_n_/I_o_) of hemeproteins versus t^1/2^ where (red) Hb, (black) Mb, and (blue) Cyt. C reacted at the MB/GP film electrode in a pH 7 buffer solution.

Using the term of I_n_/I_o_, the catalytic reaction of the hemeprotein of the MB/GP modified SPCE was investigated. I_n_/I_o_ = (π*k_cat_*Ct)^1/2^ [63] was utilized for the determination of the catalytic reaction-rate constant (*k_cat_*) of MB/GP modified on SPCE, where I_n_/I_o_ is the catalytic ratio, t is the range of reduction potential (−0.6 V to −0.1 V, approximated as 0.5 V), π = 3.14, and C = the heme concentration (0.5 µM). The catalytic reaction-rate constants (*k_cat_*) of Hb, MB, and Cyt. C. can be determined from the slope in Figure 11 and the obtained results were 2.10 × 10^−6^ (Ms)^−1^, 1.55 × 10^−6^(Ms)^−1^, and 1.50 × 10^−6^(Ms)^−1^ for Hb, MB, and Cyt. C, respectively.

The estimation of the current peak ratio (I_n_/I_o_) was evaluated from a linear sweep voltammogram, as shown in Figure 11. Io is the background current of MB/GP in the buffer and is the catalytic current of hemeproteins. Linear regressions of I_n_/I_o_ versus the square root of the scan rate were obtained. The results suggest that the reaction is diffusion-limited for potentials larger than −0.35 V and that the mechanism of the electrode reactions is an electron transfer reaction (E) that follows the chemical reaction (C) [64].

**Figure 12:**
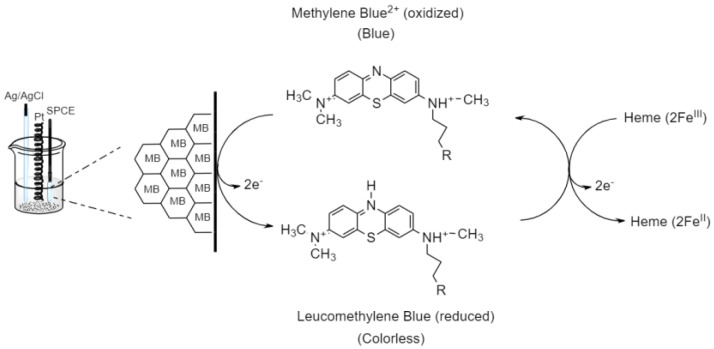
Electrocatalytic reduction of hemeproteins at MB/GP modified electrode.

Figure 12 shows the EC process which was explained with the forward scan of voltammogram. The first step is the reduction of MB at the electrode surface (inner Helmholtz plane (IHP)). Oxidized MB received two electrons at the IHP zone, thus producing its reduced form. The second step is a chemical reaction, in which reduced MB reacted with hemeproteins (Fe^III^) to produce hemeproteins (Fe^II^) at the diffusion layer.

### 3.7. Performance of MB/GP Modified Electrode

**Figure 13:**
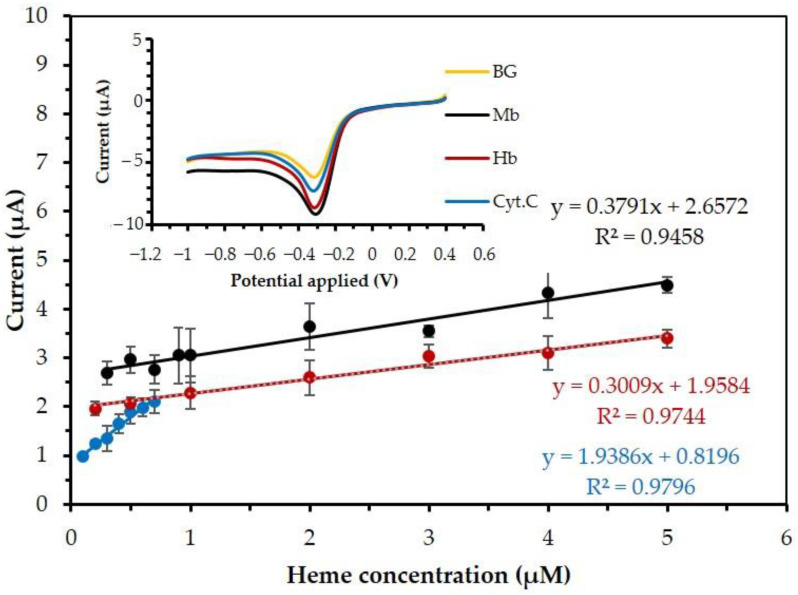
The current response of Hb (red), Mb (black), and Cyt. C (blue) vs. concentration of hemeproteins. The scan rate was used at 20 mV/s. Inset illustrates the LSV of the MB/GP-modified electrode in 0.1 M buffer solution containing 0.1 M KCl in the presence of 0.5 μM of each of Hb (red), Mb (black), and Cyt. C (blue).

The catalytic reduction peak current of hemeproteins obtained by linear sweep voltammetry (LSV) was used as the analytical signal for the determination of hemeproteins. Inset in Figure 13, the MB/GP film electrode in 0.1 M phosphate buffer, pH 7 adding 0.1 M KCl is presented at the various concentrations of hemeproteins.

Table 1 shows that the current response of hemeproteins that is linear in the range of 0.2 μM to 5 μM, 0.3 μM to 5 μM, and 0.1 μM to 0.7 μM for Hb, Mb, and Cyt. C, respectively. The detection limits were 0.2 μM, 0.3 μM, and 0.1 μM for Hb, Mb, and Cyt. C, respectively. This sensor showed excellent analytical performance. The good sensitivity can promote the electron transfer between hemeproteins and the surface of the MB/GP-modified electrode. The relation of the catalytic current ratio is explained and compared in Table 2.

Table 2 shows that the electroactive ability increased (current ratio of I_n_/I_o_) after hemeproteins were added, as shown in Figure 11. The relationship between the catalytic reaction rate constant and sensitivity depends on the structure molecule of hemeproteins. The sensitivity for Hb and Mb is lower than that of Cyt. C because Hb has four polypeptide chains. A larger molecule can produce steric which might prevent the chemical reaction between MB_red_ and Hb. For this reason, the sensitivity decreased with the number of polypeptide chains. Meanwhile, the *k_cat_* increased with the number of hemes (Fe^3+^) since 4 heme (4Fe^3+^) of Hb has the opportunity to react with MB_red_. This explains why the *k_cat_* of Hb was higher than Mb and Cyt. C.

### 3.8. Storage Stability of MB/GP Film on SPCE

The number of waiting days was measured to reduce peak current intensities, as shown in Table 3. The results show that the peak current intensities were still stable 30 days after the day of modification. Aromatic redox compounds can be adsorbed strongly onto GP due to pi-stacking interactions with the graphene sheet [50]. Therefore, the MP/GP nanohybrid electrodes show good storage stability over time.

## 4. Conclusions

A simple method was applied to construct a nanohybrid film of MB/GP that strongly adhered to the carbon-screen-printed electrode surface. It showed good stability even after 30 days of fabrication. The results show that this nanohybrid’s electrode can enhance the electron transfer of electrocatalytic reaction with hemeproteins. This study investigated the electro-catalytic reaction of an organic chemical that is present in the body as a hemeprotein of Hb, Mb, and Cyt.C. This work demonstrates the activity of modified electrodes with MB/GP nanohybrids, at which the structure of GP was attached with MB by π-π bonding interaction. The electrochemical behaviors at the modified electrode are considered suitable to learn fundamental interactions, modified electrode behaviors, and associated homogenous reactions. A heterogeneous electron transport at the electrode (E) and a subsequent homogeneous chemical reaction (C) were recognized as two mechanistic steps. This work introduced the direct heterogeneous electron transfer process, which does not require the utilization of enzyme activity. A large molecule can, however, have steric effects, as evidenced by the different catalytic ratios of each molecule. Future development can benefit from this understanding of processes.

The novel aspect of the study is the direct electro-catalytic activity measurement of hemeproteins using MB/GP nanohybrids rather than an enzyme’s catalytic activity. The developed sensor in this work is far more stable and inexpensive and can to be stored at room temperature without worrying about the enzyme’s activity deteriorating. Additionally, the MB/GP modified electrode that was developed in this work can be used as a disposable sensor for all varieties of hemeproteins. It can accurately detect the direct electro-catalytic activity of hemeproteins such as Hb, Mb, and Cyt. C. This disposable sensor has the benefit of being able to be used to diagnose a wide range of diseases because it is affordable and simple to fabricate.

## Figures and Tables

**Table 1:** The electrochemical performance of heme sensors.

Sample	Calibration Equation	Linear Range (µM)	Correlation Coefficients (R^2^)	LOD (µM)	Sensitivity (µA/µM)
Hb	Y = 0.307x + 1.95	0.2 to 5	0.98	0.2	0.307
Mb	Y = 0.363x + 2.582	0.3 to 5	0.95	0.3	0.363
Cyt. C	Y = 2.047x + 0.787	0.1 to 0.7	0.98	0.1	2.047

**Table 2:** The catalytic reaction rate constant (Figure 11) and the sensitivities (Figure 13) of hemeproteins.

Hemeproteins	The Catalytic Reaction Rate Constant (*k_cat_*, (Ms)^−1^)	Sensitivity (µA/µM)	The Structure of Hemeproteins	The 3D Feature of Hemeproteins [65,66,67]
Hb	2.10 × 10^−6^	0.307	polypeptide chains 4 heme 4 Fe ^(3+)^	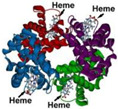
Mb	1.55 × 10^−6^	0.363	polypeptide chain 1 heme 1 Fe ^(3+)^	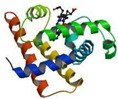
Cyt. C	1.50 × 10^−6^	2.047	polypeptide chain 1 Fe ^(3+)^ 1 heme (the smallest protein molecule of heme)	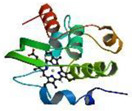

**Table 3:** The stability of MB/GP modified electrode.

The Item of the Electrode	The Number of Days	The Current Response (Ip (µA), (n = 3))
#1	1st	−4.378 (SD ± 0.720)
#2	5th	−4.137 (SD ± 0.324)
#3	10th	−4.286 (SD ± 0.538)
#4	20th	−4.511 (SD ± 0.114)
#5	30th	−4.527 (SD ± 0.290)

## Data Availability

The data are available on reasonable request from the corresponding author.
